# Smartphone‐Based Finger‐Tapping as a Predictor of Motor and Cognitive Decline in Neurodegenerative Disorders

**DOI:** 10.1002/alz70857_107319

**Published:** 2025-12-24

**Authors:** Morgan O'Connor, Mark E. Sanderson‐Cimino, Zi Li, Sreya Dhanam, Anjali Sadarangani, Joshua Downer, Ray Fregly, Amy B. Wise, Emily W. Paolillo, Hilary W. Heuer, Leah K. Forsberg, Brad F Boeve, Howard J. Rosen, John Kornak, Adam L Boxer, Adam M. Staffaroni

**Affiliations:** ^1^ University of California, San Francisco, Center for Memory and Aging, San Francisco, CA, USA; ^2^ Memory and Aging Center, UCSF Weill Institute for Neurosciences, University of California, San Francisco, San Francisco, CA, USA; ^3^ University of California, San Francisco, San Francisco, CA, USA; ^4^ Memory and Aging Center, UCSF Weill Institute for Neurosciences, University of California San Francisco, San Francisco, CA, USA; ^5^ Memory and Aging Center, Weill Institute for Neurosciences, University of California, San Francisco, San Francisco, CA, USA; ^6^ Mayo Clinic, Rochester, MN, USA; ^7^ Department of Neurology, Memory and Aging Center, University of California San Francisco, San Francisco, CA, USA; ^8^ Memory and Aging Center, Department of Neurology, Weill Institute for Neurosciences, University of California, San Francisco, San Francisco, CA, USA

## Abstract

**Background:**

Motor disturbances are common in neurodegenerative disorders. Digital health technologies can be a tool for remote, accessible, and low‐burden motor assessments. The ALLFTD Mobile App includes a finger‐tapping test (mApp‐FTT) to assess dexterity. We evaluated the reliability and construct validity of smartphone‐based measures of finger tapping in an in‐person sample.

**Methods:**

Participants (*n* = 33; mean age=68.2 [SD=8.6]) completed the mobile finger‐tapping test (mApp‐FTT) on iOS and Android devices (two counterbalanced 20‐second trials per hand) and the mechanical finger‐tapping test (FTT). The sample included cognitively unimpaired participants (22.2%) and those diagnosed with frontotemporal dementia‐spectrum disorders (41.7%), Alzheimer's dementia (22.2%), and progressive supranuclear palsy or corticobasal syndrome (13.9%). A motor domain was calculated based on neurological exam and added to the Clinical Dementia Rating® plus National Alzheimer's Coordinating Center–Frontotemporal Lobar Degeneration (CDR®+NACC‐FTLD). Pearson's correlations assessed associations between mApp‐FTT performance and FTT performance, clinical severity (CDR®+NACC‐FTLD Box Score), motor rating scales (Progressive Supranuclear Palsy Rating Scale [PSPRS] and Unified Parkinson's Disease Rating Scale [UPDRS]), and cognitive function. Due to small sample size, PSPRS subdomains were dichotomized, and t‐tests were performed to evaluate group differences based on symptom presence.

**Results:**

Total mApp‐FTT taps on iOS and Android were strongly correlated (*r* = 0.86, 95% CI [0.76, 0.92], *p* < .001). mApp‐FTT was associated with manual FTT on iOS (*r* = 0.70, 95% CI [0.52, 0.82], *p* < .001) and Android (*r* = 0.62, 95% CI [0.41, 0.77], *p* < .001). Among iOS users, total PSPRS score was negatively correlated with mApp‐FTT (*r* = ‐0.60, 95% CI [‐0.75, ‐0.39], *p* < .001). Worse mApp‐FTT performance was observed in participants with higher limb rigidity (mean difference=15.46, 95% CI [3.40, 27.52], *p* < .001), limb dystonia (mean difference=19.22, 95% CI [5.67, 32.77], *p* < .001), and finger tapping (mean difference=12.74, 95% CI [3.42, 22.06], *p* < .001). Total UPDRS score correlated with lower mApp‐FTT (*r* = ‐0.48, 95% CI [‐0.66, ‐0.28], *p* < .001), as did disease severity (CDR®+NACC‐FTLD Box Score; r=‐0.46, 95% CI [‐0.66, ‐0.22], *p* < .001).

**Conclusion:**

Smartphone‐based finger‐tapping assessments effectively detect motor and cognitive impairment, with stronger associations for motor function. Findings in iOS users highlight the need to assess operating system‐related biases and validate in Android. Remote smartphone assessments offer a scalable tool for tracking disease progression, warranting larger longitudinal studies.

